# Mapping Nucleotide Sequences that Encode Complex Binary Disease Traits with HapMap

**DOI:** 10.2174/138920207782446188

**Published:** 2007-08

**Authors:** Yuehua Cui, Wenjiang Fu, Kelian Sun, Roberto Romero, Rongling Wu

**Affiliations:** 1 Department of Statistics and Probability, Michigan State University, East Lansing, Michigan 48824; 2 Department of Epidemiology, Michigan State University, East Lansing, Michigan 48824; 3 The Perinatology Research Branch, NICHD, NIH 48201, University of Florida, Gainesville, Florida 32611, USA; 4 Department of Statistics, University of Florida, Gainesville, Florida 32611, USA

**Keywords:** Nucleotide sequence, complex disease, EM algorithm, logistic regression, haplotype.

## Abstract

Detecting the patterns of DNA sequence variants across the human genome is a crucial step for unraveling the genetic basis of complex human diseases. The human HapMap constructed by single nucleotide polymorphisms (SNPs) provides efficient sequence variation information that can speed up the discovery of genes related to common diseases. In this article, we present a generalized linear model for identifying specific nucleotide variants that encode complex human diseases. A novel approach is derived to group haplotypes to form composite diplotypes, which largely reduces the model degrees of freedom for an association test and hence increases the power when multiple SNP markers are involved. An efficient two-stage estimation procedure based on the expectation-maximization (EM) algorithm is derived to estimate parameters. Non-genetic environmental or clinical risk factors can also be fitted into the model. Computer simulations show that our model has reasonable power and type I error rate with appropriate sample size. It is also suggested through simulations that a balanced design with approximately equal number of cases and controls should be preferred to maintain small estimation bias and reasonable testing power. To illustrate the utility, we apply the method to a genetic association study of large for gestational age (LGA) neonates. The model provides a powerful tool for elucidating the genetic basis of complex binary diseases.

## INTRODUCTION

Single nucleotide polymorphisms (SNPs) are the most common genomic variations. Detecting the patterns of DNA sequence variants across the human genome, particularly the patterns of haplotypes, is a crucial step for unravelling the genetic basis of complex human diseases. With the growing density of SNP data produced by the human HapMap project [1,2], association study has been received increasing attention in the most recent years. It provides a more efficient and powerful way for disease gene discovery than traditional linkage methods [3].

The population-based case-control study is a classical method for genetic association mapping and has been widely applied to disease gene mapping with SNP data collected from unrelated individuals. The case-control design has substantial practical advantages over a family-based design given the fact that it is often difficult to collect DNA samples from relatives of affected individuals, especially for late-onset diseases. In case-control studies, disease-gene association is usually tested by focusing on one single SNP at a time using a simple *χ*^2^ test by comparing SNP allele frequencies between cases and controls [4]. The *χ*^2^ test is a detection test rather than an estimation test since it does not provide estimates of genetic effects. An alternative approach is to apply the logistic regression which can test association and estimate genetic effects while adjusting for other covariates effects.

It is well known that many human diseases are complex, which potentially involve multiple disease loci jointly functioning to give rise to an affected individual. In general, the disease status is a result of additive or multiplicative effects of many disease predisposing alleles each having a relatively small effect [5]. Methods that test each locus separately, hence, is inefficient to detect the disease-gene association. Moreover, a significant SNP allele identified by a single SNP test may not be the causal mutation for the disease, but rather shows a significant association due to linkage disequilibrium with a causal mutation [6]. A more natural approach would be to understand the genetic basis of disease status by analyzing a group of SNPs simultaneously through haplotype analysis. The advantage of haplotype inference on disease gene mapping over a single-locus approach has been shown in several studies [7-9]. Biological evidences also confirmed the importance of haplotype analysis. For example, studies showed that the alignment of multiple functional alleles along a chromosome might have great effects on a disease status, where alleles in *cis* position (as a halpotype) within a gene can function jointly to make a “super allele” with a large effect on disease phenotypes [10]. These statistical and biological evidences underscore the importance of haplotype association mapping.

Precise haplotype inference relies on complete haplotype information available for an individual. When linkage phase is ambiguous (i.e., more than one heterozygote sites), however, direct analysis by assuming known haplotypes is infeasible. A number of statistical approaches have been proposed to estimate haplotypes in unrelated individuals (e.g. [11,12]). With estimated haplotype frequencies, association can be detected by a comparison of haplotype frequencies between affected and unaffected individuals [13]. Again, this is a detection test, and hence, does not provide inference on specific haplotype effects. Others considered haplotype effects by including possible haplotypes constructed for each individual as independent variables in a generalized linear regression model setting [10,14-18], and hence ignored the interactions of haplotypes inherited from both parents. Moreover, when there are many haplotypes fitted in the model, these approaches could be suffered from potential power loss with large number of degree of freedoms.

More recently, Liu *et al*. [19] proposed a statistical approach for identifying the distinction of haplotypes and estimating haplotype effects on a quantitatively inherited trait based on the structural and organizational patterns of nucleotide sequences in the human genome [20,21]. This approach allows the characterization of DNA sequence variants that encode quantitative variation, rather than of coarse chromosomal segments as detected by conventional linkage mapping. To generalize this approach to dichotomous disease trait, in this article, we propose a statistical mapping approach based on the information provided by HapMap project to test disease-gene association adjusting for the effects of clinical risk factors. We construct a weighted prospective likelihood function with weights modelled as a function of relative diplotype frequencies. For an individual with unknown phase, the disease trait density function is modelled as a mixture distribution with mixture proportion modeled as a function of haplotype frequencies. To reduce the model degrees of freedom for an association test, we regroup haplotypes to form three composite diplotypes regardless the number of SNP loci involved. By hypothesizing one particular haplotype as the risk haplotype, we can do a systematic model selection and hypothesis test to detect DNA sequence variants, called binary trait nucleotides (BTNs), associated with the phenotypic variation of a binary disease trait. BTNs identified by this approach are biologically more meaningful than traditional mapping approaches aimed to detect quantitative trait loci [22-23].

We develop a two-stage estimation procedure to estimate parameters. Model selection criterion such as AIC is used to select the risk haplotype. Our model is developed in the maximum likelihood context and implemented with the EM and Newton-Raphson algorithm. It allows for adjustment of nongenetic covariates, such as environmental and clinical risk factors, which may provide critical information for detecting disease-gene association. Extensive simulation studies are performed to investigate the statistical behaviors of the model. Specifically, we evaluate the effect of sample size, gene action modes and sampling design on the precision of parameter estimation, testing power and type I error rate. A real example of a study of large for gestational age (LGA) neonates is applied to show the application of the model, in which significant BTNs are detected in association with LGA.

## METHODS

### Definitions and Notations 

Binary trait nucleotides (BTNs) are defined as DNA sequence variants where there exists a distinct haplotype, termed as “risk” haplotype, associated with a binary disease trait. The biological foundation of the current BTN mapping approach is built upon the haplotypes constructed with haplotype tagging SNPs (htSNPs) located within each haplotype block. Due to strong linkage disequilibrium (LD) and low haplotype diversity within each block, a small fraction of htSNPs could explain a large portion of haplotype diversity [20,21,25]. These representative htSNPs greatly facilitate genetic association study with reduced cost and improved statistical testing power. A number of algorithms has been developed for the identification of htSNPs [26-28].

Assume a sample of *n* unrelated individuals collected from a population with *n*_1_ affected (cases) and *n*_2_ unaffected (controls). In this sample, one or more candidate genes are selected based on prior knowledge. A number of SNPs are then genotyped for each candidate gene. In the current study, our interest is to search for the pattern of BTNs that are associated with a complex disease. To demonstrate the idea of BTN mapping, we first begin with a simple model containing only two htSNPs (2-SNP BTN model). A generalization for multiple SNPs is given later.

Consider two htSNPs within a haplotype block that cosegregate with the linkage disequilibrium *D* in the population. Each SNP contains two alleles denoted as 1 or 2. Let  and $p_{1}^{\left(1\right)}$ and $p_{2}^{\left(1\right)}$ be the frequencies of alleles 1 and 2 respectively at SNP 1, and $p_{1}^{\left(2\right)}$ and $p_{2}^{\left(2\right)}$ be the frequencies of alleles 1 and 2 respectively at SNP 2. $p_{1}^{\left(k\right)}+p_{2}^{\left(k\right)}=1$ for *k* = 1,2. Here we use the superscript number for SNP index and the subscript for allele index within a SNP. Random combination of these two SNPs form 4 possible haplotypes denoted as [11], [12], [21] and [22]. Their haplotype frequencies are expressed as

(1) $$p_{r_{1}r_{2}}=p_{r_{1}}^{\left(1\right)}p_{r_{2}}^{\left(2\right)}+{\left(-1\right)}^{r_{1}+r_{2}}D,r_{i}=1\text{or 2}\left(i=1,2\right)$$

where *r_1_*,*r_2_* denote the alleles of the two SNPs, respectively, and $\sum_{r_{1}=1}^{2}\sum_{r_{2}=1}^{2}p_{r_{1}r_{2}}=1$Once haplotype frequencies are estimated, allelic frequencies and LD can be obtained by solving Equation (1).

Random combination of the four maternal and paternal haplotypes forms nine observable genotypes (*G* ) denoted as 11/11,⋅⋅⋅,12/12,⋅⋅⋅22/22. The double heterozygotic genotype 12/12 contains two possible distinct diplotypes [11][22] and [12][21], and hence is phase ambiguous. The other eight genotypes are phase-known. Each diplotype contains two distinct haplotypes. Totally, there are 10 distinct phase-known diplotypes expressed as $\left[11\right]\left[11\right],\left[11\right]\left[12\right],\cdot \cdot \cdot ,\left[22\right]\left[22\right]$ formed by two SNPs. Let $P_{\left[r_{1}r_{2}\right]\left[r_{1}r_{2}\right]}$ and $P_{r_{1}r_{1}/r_{2}r_{2}}$ denote the diplotype and genotype frequencies, respectively, and let $n_{r_{1}r_{1}/r_{2}r_{2}}$ denote the number of observations of the above nine genotypes, where  *r_j_* = 1 or 2 ( *j *= 1,2). We use upper case *P* to denote the diplotype frequency and lower case *p* to denote the haplotype frequency. Assuming HWE, then ten diplotype frequencies can be calculated as a function of the corresponding haplotype frequencies, i.e., $P_{\left[r_{1}r_{2}\right]\left[r_{1}r_{2}\right]}=p_{r_{1}r_{2}}p_{r_{1}r_{2}}.$ A complete list of the genotype and diplotype configurations as well as their frequencies is given in Table ** 1**.

Without loss of generality, we assume that a disease predisposing BTN containing haplotype [11] is associated with the disease phenotype. Such a distinct haplotype [11] is called the “risk” haplotype. Individuals carrying this specific haplotype may potentially have high or low risk to develop a disease with a risk level depending on the composition of the diplotype structure one carries on. All the other three haplotypes are called non-risk haplotypes. To distinguish the risk and non-risk haplotypes, we denote all the non-risk haplotypes as $\left[\bar{11}\right]$. Random combination of these risk and non-risk haplotypes leads to three groupings which are called *composite diplotypes (g)* expressed as [11][11], [11] $\left[\bar{11}\right]$ and $\left[\bar{11}\right]\left[\bar{11}\right]$ (Table **1**). 

The regrouping method is biologically intuitive and statistically efficient. By formulating the composite diplotype, the additive and dominant effects of a risk haplotype can be estimated. Also, we could greatly reduce the number of parameters in the regression model. For example, when there are *m* SNPs considered, there could be 2*m* haplotype parameters need to be estimated for a full haplotype regression model and 2^*m*-1^(2*^m^*+1) parameters need to be estimated for a full diplotype model. When *m* is large, this could cause over-fitting problems. Moreover, large number of degree of freedom could decrease the power for an association test. With our formulation, there are always three composite diplotypes regardless of large number of SNPs. 

### Multiple Logistic Regression Model

Let *y *denote a measured disease trait which takes two values, 1 or 0, corresponding to affected or control respectively. Let *X_g_* denote a matrix of numerical codes corresponding to the composite diplotype, *g*, including the intercept as the first column, and let *X_e_ d*enote a matrix of measured clinical risk factors. Assuming that all these covariates influence the mean of the trait and not the scale, so that their effects can be summarized by a function of linear predictors

(2) $$\eta =X_{g}\alpha +X_{e}\gamma =X\beta$$

where α contain regression parameters for the intercept and the genetic effects of composite diplotypes on a disease trait; γ  contain the effects of clinical risk factors; *X* = (*X_g_*, *X_e_*) and β = (α, γ) Given a binary disease response, we can apply the logit model which corresponds to the natural logit link function with the form

$\mathrm{logit}\left(\pi \right)=\log\left(\frac{\pi }{1-\pi }\right)=\eta$

with the logistic distribution function

$\pi =h\left(\eta \right)=\frac{\exp\left(\eta \right)}{1+\exp\left(\eta \right)}$

A logistic regression model has been broadly applied to the modelling of binary data [29,30]. Given covariates value *x*, the probability distribution of a disease status  *Y* = *y* for an individual *i* can be expressed as

(3) $$\pi \left(y_{i}|x_{\mathrm{gi}},x_{\mathrm{ei}}\right)=\frac{\exp\left\{\left(\sum_{j'=0}^{2}\alpha_{j^{\prime }}x_{{\mathrm{gij}}^{\prime }}+\sum_{j=1}^{p}\gamma_{j}x_{\mathrm{eij}}\right)y_{i}\right\}}{1+\exp\left(\sum_{j'=0}^{2}\alpha_{j^{\prime }}x_{{\mathrm{gij}}^{\prime }}+\sum_{j=1}^{p}\gamma_{j}x_{\mathrm{eij}}\right)},i=1,\cdot \cdot \cdot ,n$$

where *y_i_* takes value 1 or 0, *x_gi_*_0_ is one for all *i*, the independent variables* x_gi_*_1_  and *x_gi_*_2_ are defined as

(4) $$x_{\text{gi}1}=\left\{\begin{array}{c} 1\text{ for composite diplotype}\left[11\right]\left[11\right]\\ 0\text{ for composite diplotype}\left[11\right]\left[\bar{11}\right]\\ -1\text{ for composite diplotype}\left[\bar{11}\right]\left[\bar{11}\right] \end{array}\right.$$

and

(5) $$x_{\mathrm{gi2}}=\left\{\begin{array}{c} 1\text{ for composite diplotype}\left[11\right]\left[\bar{11}\right]\\ 0\text{ otherwise} \end{array}\right.$$

and variables $x_{\mathrm{eij}}=\left(j=1,\cdot \cdot \cdot ,p\right)$ refers to the *p* clinical non-genetic covariates of interest.

With the coding mechanism defined in (4) and (5), *α*_1_ and *α*_2_ can be considered as the additive and dominant genetic effect of a risk haplotype [31], α_0_  is the intercept and $\gamma_{j}\left(j=1,\cdot \cdot \cdot ,p\right)$ is the non-genetic covariate effect. If either parameter estimate, *α*_1_ or *α*_2_, is positive, the risk haplotype [11] triggers a positive effect to increase a disease risk. The effect of BTNs is considered as pure additive, dominant or recessive if the ratio of the dominant over additive effect (*α*_2_ / α_1_) is 0, 1 or -1 respectively, and is considered as semi-dominant or over-dominant if the absolute value of this ratio is less than 1, or greater than 1 respectively. We call parameters contained in β = (α, γ ) quantitative parameters to distinguish them with the population parameters defined in Eq. (1).

We can further partition the logistic function defined in (3) into three distinct logistic regression functions corresponding to different composite diplotype groups as follows

(6) $$\pi_{2}=\pi_{2}\left(y_{i}|x_{\mathrm{gi}},x_{\mathrm{ei}}\right)=\frac{\exp\left\{\left(\alpha_{0}+\alpha_{1}+\sum_{j=1}^{p}\gamma_{j}x_{\mathrm{eij}}\right)y_{i}\right\}}{1+\exp\left(\alpha_{0}+\alpha_{1}+\sum_{j=1}^{p}\gamma_{j}x_{\mathrm{eij}}\right)}$$

for composite diplotype [11][11], and

(7) $$\pi_{1}=\pi_{1}\left(y_{i}|x_{\mathrm{gi}},x_{\mathrm{ei}}\right)=\frac{\exp\left\{\left(\alpha_{0}+\alpha_{2}+\sum_{j=1}^{p}\gamma_{j}x_{\mathrm{eij}}\right)y_{i}\right\}}{1+\exp\left(\alpha_{0}+\alpha_{2}+\sum_{j=1}^{p}\gamma_{j}x_{\mathrm{eij}}\right)}$$

for composite diplotype [11] $\left[\bar{11}\right]$, and

(8) $$\pi_{0}=\pi_{0}\left(y_{i}|x_{\mathrm{gi}},x_{\mathrm{ei}}\right)=\frac{\exp\left\{\left(\alpha_{0}-\alpha_{1}+\sum_{j=1}^{p}\gamma_{j}x_{\mathrm{eij}}\right)y_{i}\right\}}{1+\exp\left(\alpha_{0}-\alpha_{1}+\sum_{j=1}^{p}\gamma_{j}x_{\mathrm{eij}}\right)}$$

for composite diplotype $\left[\bar{11}\right]\left[\bar{11}\right]$.

We define these three distinct logistic functions as the diplotype functions corresponding to different diplotypes illustrated in Table ** 1**.

### Likelihood Function and Parameter Estimation

The logistic regression model links the interpatient variation in a disease trait (*y*) with the observed SNP genotypes (*G*). Our goal is to detect DNA sequence variants or BTNs underlying a disease trait. As shown in Table ** 1**, most genotypes have one to one relationship with their diplotypes except the one with genotype denoted as 12/12. This double heterozygote can be partitioned into two possible diplotypes, [11][22] and [12][12] with relative frequencies *ø* and 1 - *ø*, respectively. Let *p*(*g_i_* | *G_i _*) denote the relative frequency for a diplotype *g_i_* consistent with the observed genotype *G*_i_. The relative frequencies for all 10 possible diplotypes are given in Table ** 1**. For individuals with known phase, ( *p*(*g_i_* | *G_i _*) takes value one. The individual contribution to the likelihood is given by

$L_{i}\left(\beta \right)=\sum_{g_{i}\in D}\pi \left(y_{i}|x_{i}\right)p\left(g_{i}|G_{i}\right)$

where *D* denotes all possible diplotypes that are consistent with the observed marker genotype. For an individual with known phase, *L_i_*(β) = *π* (*y_i_* | *x_i_*). For an individual with genotype 12/12, its likelihood contribution follows a mixture distribution with the form

(9) $$L_{i}\left(\beta \right)=\sum_{g_{i}\in D}\pi \left(y_{i}|x_{i}\right)p\left(g_{i}|G_{i}\right)=\phi \pi_{1}\left(y_{i}|x_{i}\right)+\left(1-\phi \right)\pi_{0}\left(y_{i}|x_{i}\right)$$

where the mixture proportion

$\phi =\frac{p_{11}p_{22}}{p_{11}p_{22}+p_{12}p_{12}}$

represents the relative frequency of subject *i* whose diplotype is [11][22], and $\pi_{1}\left(y_{i}|x_{i}\right)$ and $\pi_{0}\left(y_{i}|x_{i}\right)$ are the logistic regression functions defined in model (7) and (8), respectively.

Assuming independence among individuals, the joint prospective likelihood function can be expressed as

(10) $$L\left(\beta \right)=\prod_{i=1}^{n}L_{i}\left(\beta \right)$$

Noted that the likelihood formulation in (10) is different from the one proposed by Lake *et al*. [15] in which the likelihood function is given as a weighted sum with weights modeled as a function of haplotype frequencies rather than relative diplotype frequencies.

For a 2-SNP model, the log-likelihood function of the observed data can be further partitioned as

(11) $$l_{n}\left(\beta \right)=\text{log}L\left(\beta \right)=\sum_{i=1}^{n_{11/11}}\text{log}\pi_{2}\left(y_{i}|x_{i}\right)+\sum_{i=1}^{n_{11/12}+n_{12/11}}\text{log}\pi_{1}\left(y_{i}|x_{i}\right)+\sum_{i=1}^{n_{11/22}+n_{12/22}+n_{22/11}+n_{22/12}+n_{22/22}}\text{log}\pi_{0}\left(y_{i}|x_{i}\right)+\sum_{i=1}^{n_{12/12}}\text{log}\left[\phi \pi_{1}\left(y_{i}|x_{i}\right)+\left(1-\phi \right)\pi_{0}\left(y_{i}|x_{i}\right)\right]$$

The maximum likelihood estimate ${\hat{\beta }}_{j}\left(j=0,\cdot \cdot \cdot ,p+2\right)$ contained in β can be obtained by solving the score equation: $\partial \ell_{n}\left(\beta \right)/\partial \beta_{j}=0$. A computational algorithm based on the Expectation-Maximization (EM) algorithm [32] can be formulated to find $\hat{\beta }$ , with the Newton-Raphson algorithm embedded in the M-step (See Appendix for detailed derivations). Standard model diagnostic approaches such as goodness-of-fit test can be applied to check the model fitting [33]. We array this set of quantitative parameters which include genetic and nongenetic parameters based on Model (3) as Ω_*q*_ = (β) = (α, γ ).

The above algorithm is implemented assuming that *ø* is known. In reality, we do not know *ø* and it needs to be estimated from the data. To estimate *ø*, we need to estimate the four haplotype frequencies which is arrayed as $\Omega_{p}=\left(p_{11},p_{12},p_{21},p_{22}\right)$. Once we estimate 	Ω _p_, *ø*  can be estimated by plugging in the MLE of Ω _p_.

The four haplotype frequencies can be estimated based on the nine observed genotypes $\left(\Gamma \right)$ for two SNPs (Table ** 1**). Assuming HWE, the log-likelihood function of the unknown haplotype frequencies given observed genotypes can be written as a multinomial distribution 

$\begin{array}{c} \text{log}L\left(\Omega_{p}|G\right)\alpha \\ \begin{array}{c} 2n_{11/11}\text{log}p_{11}+n_{11/12}\text{log}\left(2p_{11}p_{12}\right)+2n_{12/12}\text{log}p_{12}\\ +n_{12/11}\text{log}\left(2p_{11}p_{21}\right)+n_{12/12}\text{log}\left[2\left(p_{11}p_{22}+p_{12}p_{21}\right)\right]\\ +n_{12/22}\text{log}\left(2p_{12}p_{22}\right)+{\text{2n}}_{21/21}\text{log}p_{21}+n_{21/22}\text{log}\left(2p_{21}p_{22}\right)\\ +{\text{2n}}_{22/22}\text{log}p_{22} \end{array} \end{array}$

Again, we have a missing data problem since the two distinct diplotypes for genotype 12/12 can not be observed explicitly. This problem can be solved by applying the EM algorithm (See [19] for a detailed EM procedure). With the estimated haplotype frequencies, we can also solve Equation (1) to obtain the estimates of the SNP allele frequencies and the LD parameter.

The estimated *ø*, denoted as $\hat{\phi }$, is then plugged into the likelihood function (11) to obtain the parameter estimation contained in  Ω_*q*_ . Since we estimate parameters contained in  Ω_*q*_  and  Ω_*p*_  separately, this estimation procedure is also called a two-stage estimation procedure. Noted that the parameters contained in  Ω_*q*_  do not heavily rely on the estimated haplotype frequencies, especially when the double heterozygous rate is low. Thus, the estimation procedure is quite robust to departure from HWE. Both EM algorithms for estimating  Ω_*q*_ and Ω_*p*_ converge very fast.

### Hypothesis Tests

To detect the association between a disease and BTNs and fully dissect the genetic effects of BTNs, a series of hypotheses can be conducted. The existence of significant BTNs on a complex disease trait can be tested based on the following hypotheses

(12) $$\left\{\begin{array}{c} H_{0}:\alpha_{1}=\alpha_{2}=0\\ H_{1}:\text{at least one of the parameters doesnot equal 0} \end{array}\right.$$

A general approach is to use the likelihood ratio test, where the test statistic is calculated by comparing the likelihood values under the alternative hypothesis *H_1_* to the null hypothesis *H_0_* for the significance of BTNs using 

$\text{L}R_{1}=-2\left[\text{log}L\left({\tilde{\alpha }}_{0},\tilde{\gamma },\alpha_{1}=\alpha_{2}=0|G\right)-\text{log}L\left(\hat{\alpha },\hat{\gamma }|G\right)\right]$

where the parameters with tilde and hat denote the MLEs of unknown parameters under *H*_0_ and  *H*_1_, respectively. Assuming fixed *ø* in the likelihood function (11), the regularity conditions for asymptotic *χ*^2^ distribution of LR_1_ hold as long as the number of observations *n*_11/12_ + *n*_12/11_ and *n*_11/22_ + *n*_12/22_ + *n*_22/11_ + *n*_22/12_ + *n*_22/22_ are of comparable size with *n*_12/12_. So the LR_1_ asymptotically follows a *χ*^2^ distribution with two degrees of freedom [34].

Upon rejection of *H_0_* in the above test, we can further test whether the BTNs exert a significant additive haplotype effect or dominant haplotype effect on a disease trait by simply formulating

(13) $$\left\{\begin{array}{c} H_{0}:\alpha_{1}=0\\ H_{1}:\alpha_{1}\neq 0 \end{array}\right.$$

for testing additive effect and

(14) $$\left\{\begin{array}{c} H_{0}:\alpha_{2}=0\\ H_{1}:\alpha_{2}\neq 0 \end{array}\right.$$

for testing dominant effect.

Again, the likelihood ratio test can be applied which is asymptotically *χ*^2^ -distributed with one degree of freedom.

We can also test the allelic association between two SNPs by testing the LD between them with hypotheses:

(15) $$\left\{\begin{array}{c} H_{0}:D=0\\ H_{1}:D\neq 0 \end{array}\right.$$

The log-likelihood ratio test statistic (LR_2_) can be similarly calculated as 

$LR_{2}=-2\left[\text{log}L\left({\tilde{p}}_{1}^{\left(1\right)},{\tilde{p}}_{1}^{\left(2\right)},D=0|G\right)-\text{log}L\left({\hat{\Omega }}_{p}|G\right)\right]$

The LR_2_ is considered to asymptotically follow a *χ*^2^ distribution with one degree of freedom. The MLEs of allelic frequencies under  *H*_0_ can be estimated using the EM algorithm described above, but with the constraint *p*_11_*p*_22_ = *p*_12_*p*_21_.

The effect of non-genetic covariates on a disease trait can also be tested in a similar way using likelihood ratio test. Since the association test (12) is conducted after adjusting for the effects of clinical risk factors, it is more informative than the retrospective likelihood approaches (e.g. [14,16]) which do not adjust for the effects of clinical risk factors.

### Risk Haplotype Selection and Statistical Inferences

The above model is developed by assuming that haplotype [11] is the risk haplotype. In reality, we have no prior information on which genetic component triggers a potential effect on a disease trait. We adopt the theoretical information criterion approach to select the risk haplotype. Among a pool of criteria, the Akaike's information criteria (AIC) has been widely used in a variety of fields for model selection [35]. For a 2-SNP model, there are 4 possible haplotype structures. By assuming each one of the haplotypes as the risk haplotype, we can calculate the AIC information one at a time for each hypothesized risk haplotype as

(16) $$\text{AIC}=-\text{2 ln}L\left(\beta |s\right)+2p_{s}$$

where *s* refers to the *s*th haplotype and *p_s_* refers to the number of parameters by taking the *s*th haplotype as the risk haplotype. The one which achieves the minimum AIC value is then subject to statistical test based on test (12). Significant BTNs are detected to be associated with a disease if a significant risk haplotype exists. When there are multiple haplotype blocks involved, corrections for multiple testing using false discovery rate (FDR) approach is required [36].

A number of statistical inferences can be formulated based on the current BTN model. If significant BTNs are detected, one might be interested in quantifying the disease odds or odds ratio. The disease odds can be calculated for individuals carrying different haplotype structures and are exposed to different clinical conditions. For example, the odds of a disease for an individual carrying composite diplotype [11][11] can be calculated as 

${\mathrm{odds}}_{\left[11\right]\left[11\right]}=\frac{p\left(y_{i}=1|X_{\mathrm{gi},}X_{\mathrm{ei}}\right)}{p\left(y_{i}=0|X_{\mathrm{gi},}X_{\mathrm{ei}}\right)}=\exp\left(\alpha_{0}+\alpha_{1}+\sum_{j=1}^{p}\gamma_{j}x_{\mathrm{eij}}\right)$

Thus the exponential of the parameters gives rise to a factorial contribution to the odds not only subject to clinical exposure but also to diplotype structure. Even though individuals are exposed to the same clinical condition, the chance to be affected varies depending on the diplotype structures they carry on. For example, the odds ratio of a disease for an individual carrying composite diplotype [11][11] and $\left[\bar{11}\right]\left[\bar{11}\right]$after controlling for other covariates can be computed as 

$OR_{\left[11\right]\left[11\right]/\left[\bar{11}\right]\left[\bar{11}\right]}=\frac{p\left(y_{i}=1|\;X_{\mathrm{g2i}}=1,X_{\mathrm{ei}}\right)/p\left(y_{i}=0|X_{\mathrm{g2i}}=1,X_{\mathrm{ei}}\right)}{p\left(y_{i}=1|X_{\mathrm{g2i}}=-1,X_{\mathrm{ei}}\right)/p\left(y_{i}=0|X_{\mathrm{g2i}}=-1,X_{\mathrm{ei}}\right)}=\exp\left(2\alpha_{1}\right)$

Using delta method, the confidence interval of the odds ratio can be obtained [33]. Note that the intercept *α*_0_ does not represent population prevalence for a case-control sample.

### Multilocus BTN Model

The idea of BTN mapping based on a two-SNP model can be extended to include an arbitrary number of SNPs whose sequence variants are associated with the disease variation. Consider *K*(*K* ≥ 3) htSNPs within a haplotype block constructed from a number of bi-allelic loci. Each of these *K* htSNPs contains two alleles denoted by $Q_{r_{k}}^{k}\left(r_{k}=1,2;k=1,\cdot \cdot \cdot K\right)$, with allele frequencies denoted by $p_{r_{k}}^{\left(k\right)}$ for the *k* th htSNP. The coding form indicates that alleles with the same value of *r*_k_ are located on the same chromosome.

One of the key issues for the multi-SNPs model is to clearly formulate the haplotype and diplotype structures across the *K* multilocus htSNPs. There are totally 2^k^ possible haplotypes can be formed by the random combination of these *K* htSNPs. A general form of these haplotypes is expressed as $Q_{r_{1}}^{1}Q_{r_{2}}^{2}\cdot \cdot \cdot Q_{r_{k}}^{K}$ with corresponding haplotype frequencies denoted by $p_{r_{1}r_{2}...r_{k}}$. These *K* htSNPs form 3^k^observable multilocus zygotic genotypes expressed as 

$Q_{r_{1}}^{1}Q_{s_{1}}^{1}/Q_{r_{2}}^{2}Q_{s_{2}}^{2}/\cdot \cdot \cdot /Q_{r_{K}}^{K}Q_{s_{K}}^{K}$

with corresponding genotype frequency and observation expressed as

$P_{r_{1}s_{1}/r_{2}s_{2}{/\cdot \cdot \cdot /r}_{K}s_{K}}$

and

$n_{r_{1}s_{1}/r_{2}s_{2}/\cdot \cdot \cdot /r_{K}s_{K}}$

respectively. The random combination of haplotypes derived from maternal and paternal parents generates $2^{k-1}\left(2^{k}+1\right)$ distinct diplotypes expressed as 

$\left[Q_{r_{1}}^{1}Q_{r_{2}}^{2}\cdot \cdot \cdot Q_{r_{K}}^{K}\right]\;\left[Q_{s_{1}}^{1}Q_{s_{2}}^{2}\cdot \cdot \cdot Q_{s_{K}}^{K}\right]$

with corresponding diplotype frequency expressed as 

$P_{\left[r_{1}r_{2}\cdot \cdot \cdot r_{K}\right]\left[s_{1}s_{2}\cdot \cdot \cdot s_{K}\right]}\;=p_{r_{1}r_{2}\cdot \cdot \cdot r_{K}}p_{s_{1}s_{2}\cdot \cdot \cdot s_{K}}$

assuming HWE. The composite diplotype can be formulated in a similar way as illustrated in the 2-SNP model.

As illustrated in the 2-SNP BTN model, the number of multilocus diplotype is generally greater than the number of genotypes when there are two or more heterozygotes present. For example, for a 3-SNP model, the genotype $Q_{1}^{1}Q_{1}^{1}{/Q}_{1}^{2}Q_{2}^{2}{/Q}_{1}^{3}Q_{2}^{3}$ could form two different diplotypes expressed as $\left[Q_{1}^{1}Q_{1}^{2}Q_{1}^{3}\right]\;\left[Q_{1}^{1}Q_{2}^{2}Q_{2}^{3}\right]\text{ and }\left[Q_{1}^{1}Q_{1}^{2}Q_{2}^{3}\right]\;\left[Q_{1}^{1}Q_{2}^{2}Q_{1}^{3}\right],$ while the genotype $Q_{1}^{1}Q_{2}^{1}{/Q}_{1}^{2}Q_{2}^{2}{/Q}_{1}^{3}Q_{2}^{3}$ could form four different diplotypes. If we assume that $Q_{1}^{1}Q_{1}^{2}Q_{1}^{3}$ is the risk haplotype, the three composite diplotypes can be formulated as $\left[Q_{1}^{1}Q_{1}^{2}Q_{1}^{3}\right]\;\left[Q_{1}^{1}Q_{1}^{2}Q_{1}^{3}\right],\left[Q_{1}^{1}Q_{1}^{2}Q_{1}^{3}\right]\;\left[\bar{Q_{1}^{1}Q_{1}^{2}Q_{1}^{3}}\right]\text{and}\left[\bar{Q_{1}^{1}Q_{1}^{2}Q_{1}^{3}}\right]\;\left[\bar{Q_{1}^{1}Q_{1}^{2}Q_{1}^{3}}\right]$ .

The multilocus haplotype frequency can be formulated as a function of allele frequencies and LD parameters of different orders [37]. For example, a haplotype frequency, denoted as $P_{r_{1}r_{2}\cdot \cdot \cdot r_{L},}$, can be decomposed into the following components: 

Pr1r2...rK=pr1pr2...prK												No LD+−1rK−1+rKpr1...prK−2DK−1K+...+−1r1+r2pr3...prKD12				Digenic LD+−1rK−2+rK−1+rKpr1...prK−3DK−2K−1K+...+−1r1+r+r3pr4...prKD123		Trigenic LD+...+−1r1+...+rKD1...K											K−genic LD

where *D* ^'^s are the linkage disequilibria of different orders among particular htSNPs.

The MLEs of quantitative parameters can be estimated by formulating the likelihood function similar to the 2-SNP model. The EM algorithm can be employed to estimate the MLEs of haplotype frequencies, and the quantitative parameters. The AIC-based model selection procedure can be adopted to select the risk hapltoype.

## RESULTS

### Simulation Study

We perform a series of Monte Carlo simulations to investigate the statistical behavior of the proposed BTN mapping approach. The simulation is designed to evaluate the model performance considering the effects of sample sizes (*n* = 100,200 and 500), gene action mode (additive, dominant, and recessive), and sampling design on the precision of parameter estimations, type I error rates as well as the power to detect the association.

Assuming that one haplotype is distinct from the other ones, haplotype frequencies are calculated based on the given allele frequencies and LD parameter as listed in Table **3**. Then distinct diplotypes are simulated according to a multinomial distribution with a probability for each diplotype calculated from their corresponding haplotype frequencies assuming HWE. A disease status is simulated from a bernoulli distribution with a probability of success defined in model (3) with *y*_i_ = 1. For simplicity, we only consider one covariate in the model and it is simulated from a standard normal distribution. The given values for population and quantitative parameters are listed in Table **3**. The data simulated with this distinct BTN structure are subject to statistical analysis.

In each simulation scenario, 1000 Monte Carlo repetitions are performed. For each Monte Carlo sample, the EM algorithm is used to obtain the MLE's of the haplotype frequencies, allele frequencies and LD parameter as well as the quantitative parameters which include the genetic and non-genetic covariates effects. The MLEs for all parameters are listed in Table **3** and their square root of the mean squared errors (RMSEs) are given in the parenthesis. The proportion of cases for the simulated data is about 40-50% on average under the three gene action modes.

As expected, the true association can only be detected with the hypothesized risk haplotype, and all the parameters can be accurately estimated only under the correct haplotype distinction. Overall, our model provides reasonable parameter estimation under different simulation scenarios. All population parameters including the allele frequencies and LD parameter can be well estimated with high precision. The precision depends only on sample size and is not affected by gene action modes. Large sample size always leads to low bias and high precision (Table **3**), which infers the consistency of the parameter estimation.

With the estimated haplotype frequencies, we carry out the second stage estimation to estimate the quantitative parameters. As can be seen from Table **3**, the estimation precision of quantitative parameters depends not only on sample size, but also on gene action modes. In general, trends hold across different simulations are evident. First, the accuracy and precision of all parameter estimation increase as the sample size increases. Small sample size (*n* = 100) results in poor parameter estimation and the precision is dramatically improved when sample size is increased from 100 to 200. Second, as expected, the additive effect can be better estimated than the dominant effect in all simulations. For instance, the RMSE for additive effect is 30% smaller than the dominant effect under the dominant model with sample size 200. Third, the nongenetic covariate effect is not sensitive to the gene action mode, whereas the genetic parameters act differently under different gene action modes.

Type I error evaluation is summarized in Table **2** at the 0.05 nominal level with sample sizes ranging from 100 to 500. It can be seen that the estimated type I error rates are not appreciably different from the nominal level 0.05. For power analysis, we consider three disease models: additive, dominant, and recessive as given in Table ** 3**. The testing power is defined as the percentage of simulations in which the true association is detected. For each simulation case, we run 1000 replicates. The results show that the power of an association test statistic depends on a number of parameters, such as the sample size and the gene action modes. As expected, the testing power increases as the sample size increases. For example, assuming additive model, the power increases from 64% to 93.3% when the sample size increases from 100 to 200. Simulation results also show that the test power is sensitive to gene action mode for small sample size (*n* = 100). When sample size increases to 200, we observe dramatic power improvement and the difference among different gene action modes is no longer remarkable.

Our association test is conducted based on a prospective likelihood setting assuming a random sample from a population. To evaluate the effect of sampling design on parameter estimation and testing power, we simulate samples through changing the intercept α_0_ by holding other parameters unchanged as given in Table ** 3**. The value of intercept determines the proportion of cases in a sample. Fig. (**3**) plots the effects of case proportions on the type I error rate with sample size 100 and 200. It can be seen that the type I error rates are inflated when the proportion of cases is away from 50%. As long as the case proportion is kept within the 30%-70% range, the false positive rate can be appropriately controlled. Figs. (**1**) and (**2**) plot the effect of case proportions on the testing power and the absolute averaged bias of parameter estimation under three disease models out of 1000 simulations. Clearly, the testing power and estimation biases are affected by case proportions. The power is greatly reduced and the parameter estimation is severely biased when the case proportion is far away from 50% for small sample size (say 100). As sample size increases to 200, the power is significantly increased and the bias is dramatically reduced. Higher sample size (500) leads to more dramatic improvement (data not shown). However, to achieve desired power and small bias, we still need to maintain a balanced case-control sample. When sample size is small (say less than 100), maintaining such a balance is even more crucial.

To test the performance of multi-SNP model, a simulation study assuming 3 htSNPs in a haplotype block is performed. The simulation design is similar to the 2-SNP model. The results are summarized in Table **4**. In general, we observe similar trends for both population and quantitative parameters as in the 2-SNP model. As compared to the 2-SNP model, a slightly higher testing power is observed compared to the 2-SNP model with 100 sample size, especially under the dominant gene action mode. When sample size increases to 200 or 500, the difference is not remarkable. Similar results are observed for case proportion effect as in the 2-SNP model and hence is omitted.

### A Case Study

We apply our model to a genetic association study of LGA neonates. LGA may lead to complications for both newborns and mothers. Studies showed that LGA is associated with increased risk of infant mortality [38], and may further lead to development of overweight for a baby in later stage of life [39, 40]. Risk of mothers of LGA neonates includes prolonged labor [41], risk of postpartum bleeding and genital tract injury [42]. Increasing proportion of LGA infants born has been reported in recent years [43, 44], but the etiology of LGA remains largely unknown. It has been increasingly recognized that complication of pregnancy and delivery is a complex trait determined by multiple environmental and genetic factors [45], few genetic association studies have been reported in literature on the relationship between genetic factors and LGA.

To understand the genetic basis of LGA, a number of candidate genes have been genotyped for SNPs. Here we only use one of them to demonstrate the model implementation. Our goal is to study which genetic factors in mother are associated with the LGA neonates. The data set contains  552 unrelated maternal individuals with 117 cases and 435 controls in ages ranging from 13 to 45 years old mothers recruited at the Sotero del Rio Hospital, in Puente Alto, Chile. Each of these subjects was genotyped for SNP markers within the candidate gene apolipoprotein C-III (APOC3) located at chromosome 11*q*23. There are total 6 positions showing polymorphisms, three at the intron 1 region for SNPs 633938761, 633938806, and 633938845, one at exon 3 region for SNP 633938988, one at intron 3 region for SNP 633939053 and one at exon 4 region for SNP 633939147. We use Haploview software to construct the haploype block [46]. The haplotype block is defined using the confidence intervals definition [21]. Fig. (**4**) shows that there are two haplotype blocks with block I containing two SNPs 633938806 and 633938845 and block II containing three SNPs 633938988, 633939053 and 633939147. SNP 633938761 does not belong to any blocks. No SNPs are significant using the single SNP *x*^2^ test implemented in Haploview. Also, no haplotypes are significant in block II and one haplotype (TC) is significant in block I using the haplotype test implemented in Haploview.

We apply our newly developed method to analyze this data set. We fit the 2-SNP model for SNPs in block I and the 3-SNP model for SNPs in block II. Results show that only SNPs in block I are significantly associated with LGA. In the following, we only focus our analysis on SNPs in block I. The two SNPs in block I form four haplotypes designated as TG, TC, CG, and CC. The two SNPs are in linkage disequilibrium, which suggests the importance of considering haplotype effect on the association study, rather than based on a single SNP. Five clinical risk factors are included in the model, maternal age (MA), maternal weight (MW), number of preterm deliveries (PTD), baby sex (BS), and maternal body mass index (MBMI). Our aim is to detect haplotype variants within this candidate gene which are associated with LGA under a variety of environmental conditions.

By assuming that one haplotype is different from the rest of the haplotypes, we performed a systematic test for the four haplotypes. The results are summarized in Table ** 5**. The MLEs of the haplotype frequencies, allele frequencies and the LD parameter are given. These two SNPs are strongly associated with each other ( $\overset{\wedge }{D}=-0.1577$ ). The estimated allele frequencies are 0.7455 for allele T in SNP 633938806 and 0.3806 for allele C in SNP 633938845. The heterozygote rate for the two SNPs are 40% and 48%, respectively. The smallest AIC value is observed for haplotype TC which also shows significance based on hypotheses test (12) (p-value=0.024). All the other three haplotypes do not show evidence of significance.

The MLEs of the quantitative parameters and their standard errors in the parenthesis are listed in Table ** 5**. The likelihood ratio test shows that both additive and dominant effect for haplotype TC are significant at the 0.01 level.Among the five non-genetic covariates, variables MA and MW are significant at the 0.05 level and variable BS is significant at the 0.01 level, which indicate that both maternal age and weight could be potential risk factors for LGA. Since the additive effect is negative, this indicates that this risk haplotype TC triggers a negative effect on LGA, i.e., individuals who carry composite diplotypes $\left[\mathrm{TC}\right]\left[\bar{\mathrm{TC}}\right]\text{and}\left[\bar{\mathrm{TC}}\right]\left[\bar{\mathrm{TC}}\right]$ have higher risk to develop LGA than individuals carrying composite diplotype [TC][TC] with odds ratio ${\mathrm{OR}}_{\left[\bar{\mathrm{TC}}\right]\left[\bar{\mathrm{TC}}\right]/\left[\mathrm{TC}\right]\left[\mathrm{TC}\right]}=3.3582\text{ and }{\mathrm{OR}}_{\left[\mathrm{TC}\right]\left[\bar{\mathrm{TC}}\right]/\left[\mathrm{TC}\right]\left[\mathrm{TC}\right]}=1.6711$ by holding constant for other risk factors.

We also calculated the odds ratio of developing LGA for individuals giving birth to different sex of baby. For example, the risk for women carrying the same composite diplotypes to develop LGA would be 1.73 times higher if they deliver baby boy compared to those who deliver baby girl. The risk to develop LGA for a 40-year old mother would be 1.7 times higher than a 25-year old mother after adjusting for other covariates effects. Holding constant for other covariates, the risk to develop LGA will be increased by 1.54 times for every 22 pounds weight gain.

## DISCUSSION

The study of common diseases can be broadly divided into two categories: family-based linkage studies across the entire genome, and population-based association studies of individual candidate genes [2]. While accumulative evidences have shown that linkage methods have lower power than population-based association methods [47,48], a more efficient way to study the genetic architecture of a complex disease is at the population level. Meanwhile, a number of studies have shown that haplotype-based association study is more powerful than single SNP analysis, especially when multiple disease-susceptibility variants occur within the same gene [11,49]. Therefore, hunting for specific DNA sequence patterns that are associated with the variation of a disease would provide efficient information in understanding the disease etiology. The model presented here, aimed to detect the association between DNA sequence variants and a binary disease trait, thus prides a timely tool toward better understanding of the genetic architecture of a complex binary disease trait.

In this article, we make an attempt to study genetic association by utilizing the abundant sequence variation information developed by the HapMap project. The model, called BTN mapping, is derived on the basis of multilocus haplotype analysis using a finite number of htSNPs within a haplotype block assuming that the candidate gene is not imprinted. Both simulation studies and a real example show that our BTN mapping approach can detect haplotype association underlying a disease trait with high power and, hence displays a number of merits.

First, our approach can characterize the association of DNA sequence variants predisposing to a disease. Traditional disease mapping approaches such as binary trait locus (BTL) mapping, attempt to identify loci called BTLs that are linked with known markers [22-24]. The specific DNA sequence structure for the detected loci remains unknown. As opposed to this traditional “indirect” approach, our model can directly materialize DNA sequences underlying a disease trait, and therefore represents a “direct” approach. It should be noted that our approach is limited by knowledge about the complete functional sequence variants information in candidate regions. With the release of more SNPs by HapMap, our model will become more useful in search for causal variants throughout the whole genome.

Second, our approach is likelihood-based and is computationally fast. Regular model selection criteria such as the AIC criterion can be applied to determine the risk haplotype structures on a disease trait. The developed model also allows for nongenetic covariates effect which might provide potential information in genetic association study. It has been shown that risk of complex diseases such as cancers may be determined by both genetic and environmental factors [50]. Incorporating the non-genetic factors should provide more meaningful results in association tests, and hence should be more preferred.

Third, the proposed regrouping approach could potentially increase testing power by reducing the degree of freedom for an association test. In general, there are two ways to increase the power of an association test: developing appropriate statistical forms for an association test or reduce the degrees of freedom [51]. A common limitation for existing haplotype-based analyses is that the test statistic often involves a large number of degrees of freedom. When large number of SNP markers are involved to construct haplotypes, the test could suffer from severe power loss as the number of degrees of freedom could be large. Through regrouping haplotypes, the BTM mapping approach has better control of the degrees of freedom because the number of degrees of freedom for an association test remain the same regardless of the number of SNPs fitted in the model. Our simulation studies confirmed that even for small sample size ( < 200), when balanced sampling scheme is maintained, one can still obtain appropriate power.

Finally, the model is robust and flexible to different genetic and experimental settings. The results from simulation studies indicate that the association between haplotype and disease phenotype can be well detected under different gene action modes with modest sample size. It is worthy to note that the proportion of cases shows a great impact on testing power and parameter estimation by various simulations. Results indicate that a nearly balanced sampling design provides optimal power and low estimation biases, especially for small sample size. Therefore, a balanced case-control design should always be preferred when recruiting samples in a case-control study.

The effect of allele frequencies on testing power and parameter estimation is also investigated (data not shown). Our results indicate that testing power is not affected by allele frequency as long as the proportion of individuals carry double risk haplotypes is not terribly small. However, we do observe inflated variances for the genetic parameters when sample size or allele frequency is small, which might be due to multicollinearity among the genetic covariates given the categorical nature of variables [15]. One possible solution is to apply a penalized regression model in which the log-likelihood function is penalized by a penalty term. Possible choices for the penalty term are Lasso type penalty [52] or ridge penalty [53]. When the likelihood function is penalized, however, the usual likelihood ratio test can not be applied. Efficient and robust model selection approaches need to be developed to solve this problem.

Our model is developed based on tag SNPs selected within a haplotype block. The purpose of using htSNPs is to control the number of SNPs used to construct a haplotype, which in turn, minimizes the computation burden for haplotype construction using EM algorithm. We may lose potential information when the efficiency of tag SNP selection is low. With more and more studies focused on tag SNP selection, more robust tagging approaches will improve our mapping efficiency. It should also be noted that the assumption by using tag SNPs can be relaxed in which all SNPs identified within a haplotype block can be used for analysis, especially when the SNPs size within a block is limited. However, corrections for multiple testings are necessary when the number of tested blocks are large.

Our mapping approach considers binary disease traits and is a generalization of the model proposed by Liu *et al*. [19] which considers continuous disease traits. The specific utility of our model to a real example from a genetic association study leads to the successful detection of BTNs genotyped within the APOC3 candidate gene associated with LGA. Although our simulation studies and the example were illustrated based on the 2-SNP and 3-SNP models, our BTN mapping model has been developed to allow for the detection of BTN structures involving any number of SNPs. The model can also be easily extended to model the interaction between genetic factors and environments. It is also possible that haplotypes in one block interact with haplotypes in other blocks. A further extension of the model can be applied to model the effects of BTN-BTN interactions on a disease trait.

## Figures and Tables

**Fig. (1):**
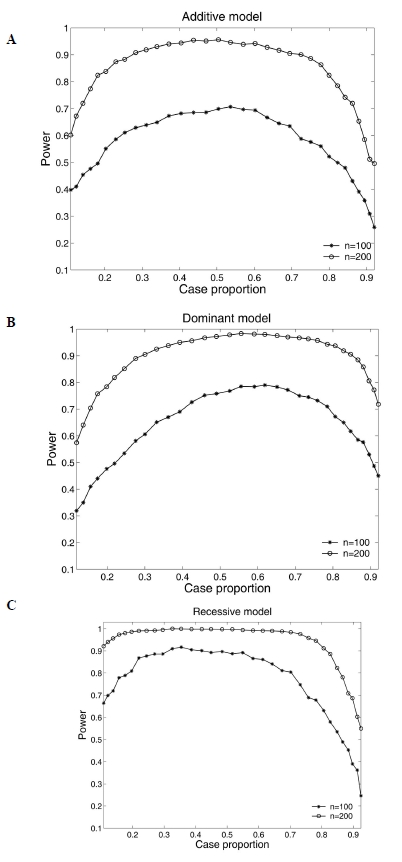
The effect of case proportion on testing power under different sample sizes under the additive model (**A**), dominant model (**B**), and recessive model (**C**). Testing power is defined as the proportion of simulations (1000 Monte Carlo simulations) in which significant associations are detected.

**Fig. (2):**
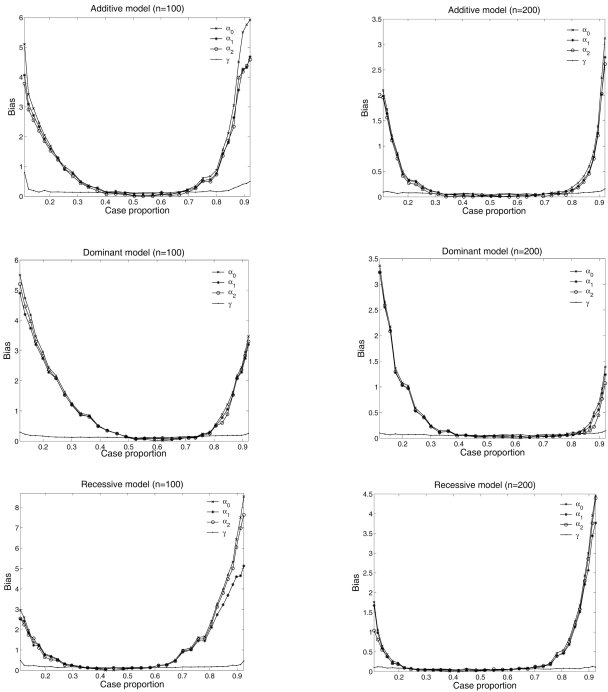
The effect of case proportion on parameter estimations under different sample sizes and different disease models. The vertical line represents the averaged absolute bias for each parameter from 1000 Monte Carlo simulations.

**Fig. (3):**
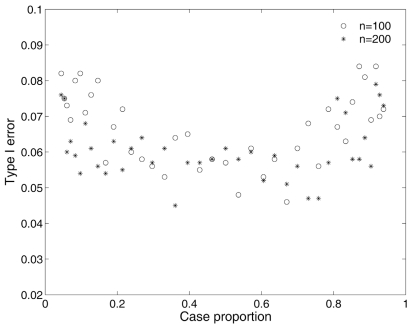
The effect of case proportion on type I error under different sample sizes. Type I error is defined as the proportion of simulations (1000 Monte Carlo simulations) in which false associations are detected with data simulated under the null of no association, i.e., *α_1_* =*α_2 _*= 0.

**Fig. (4):**
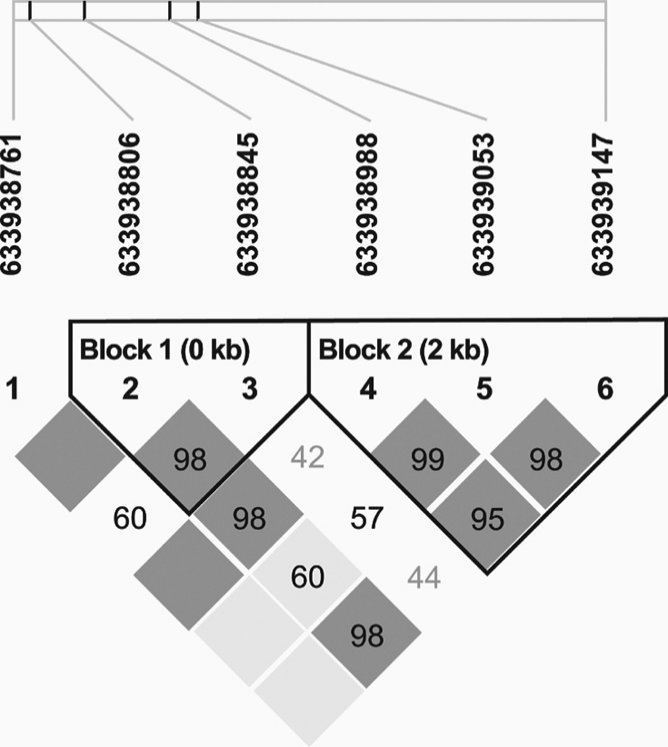
The haplotype block view constructed with Haploview [46]. The values shown on the plot are the Lewontin's D'. The blocks are defined based on the confidence intervals definition [21]. The six SNPs form two haplotype blocks with one containing two SNPs and the other one containing three SNPs. SNPs 633938761 does not belong to either of the two blocks.

**Table 1.** Possible Diplotype and Composite Diplotype Configurations of Nine Genotypes at Two SNPs and their Haplotype Composition Frequencies

Genotype	Diplotype			Composite Diplotype		No. of Observation
	Configuration	Frequency	Relative Frequency	Symbol	Diplotype Function	
11/11	[11][11]	*P*_[11][11]_ = $p_{11}^{2}$	1	[11][11]	*π*_2_	*n*_11/11_
11/12	[11][12]	*P*_[11][12]_ = 2*p*_11_*p*_12_	1	[11]$\left[\bar{11}\right]$	*π*_1_	*n*_11/12_
11/22	[12][12]	*P*_[12][12]_ = $p_{12}^{2}$	1	$\left[\bar{11}\right]$$\left[\bar{11}\right]$	*π*_0_	*n*_11/22_
12/11	[11][21]	*P*_[11][21]_ = 2*p*_11_*p*_21_	1	[11]$\left[\bar{11}\right]$	*π*_1_	*n*_12/11_
12/12	$\left\{\begin{array}{c} \left[11\right]\left[22\right]\\ \left[12\right]\left[21\right] \end{array}\right.$	$\left\{\begin{array}{c} P_{\left[11\right]\left[22\right]}={2p}_{11}p_{22}\\ P_{\left[12\right]\left[21\right]}={2p}_{12}p_{21} \end{array}\right.$	$\left\{\begin{array}{c} \phi \\ 1-\phi \end{array}\right.$	$\left\{\begin{array}{c} \left[11\right]\left[\bar{11}\right]\\ \left[\bar{11}\right]\left[\bar{11}\right] \end{array}\right.$	$\left\{\begin{array}{c} \pi_{1}\\ 1-\pi_{1} \end{array}\right.$	*n*_12/12_
12/22	[12][22]	*P*_[12][22]_ = 2*p*_12_*p*_22_	1	$\left[\bar{11}\right]$$\left[\bar{11}\right]$	*π*_0_	*n*_12/22_
22/11	[21][21]	*P*_[21][21]_ = $p_{21}^{2}$	1	$\left[\bar{11}\right]$$\left[\bar{11}\right]$	*π*_0_	*n*_22/11_
22/12	[21][22]	*P*_[21][22]_ = 2*p*_21_*p*_22_	1	$\left[\bar{11}\right]$$\left[\bar{11}\right]$	*π*_0_	*n*_22/12_
22/22	[22][22]	*P*_[22][22]_ = $p_{22}^{2}$	1	$\left[\bar{11}\right]$$\left[\bar{11}\right]$	*π*_0_	*n*_22/22_

$\phi =\frac{p_{11}p_{22}}{p_{11}p_{22}+p_{12}p_{21}}$ where *p*_11 _, * p*_12 _, * p*_21_ and *p*_22_ are the frequencies for haplotype [11], 12, 21, and 22, respectively. The relative frequency refers to the probability that a specific diplotype is observed. For unambiguous genotype (phase known), the relative frequency is 1. For the double heterozygotic genotype 12/12, the probability of observing diplotype [11][22] is *ø* , and observing diplotype [12][12] is 1- *ø* .

**Table 2.** The Type I Error Estimated from 1000 Simulation Replicates Under the 2 and 3-SNP Models with Nominal Level 0.05

n	2-SNP model	3-SNP Model
100	0.073	0.06
200	0.056	0.055
300	0.058	0.054
400	0.045	0.049
500	0.047	0.048

**Table 3.** The Mean MLEs with their Square Root Mean Square Errors (RMSEs) (in Parentheses) of Population and Quantitative Parameters of the BTNs Estimated from 1000 Simulation Replicates Under the 2-SNP Model

*n*	*α*_0_ = 0.5	*α*_1_ = 1	*α*_2_	*γ * = 1.5	$p_{1}^{\left(1\right)}=0.7$	$p_{1}^{\left(2\right)}=0.7$	*D* = 0.02	Power
**Additive**			***α *_2_= 0**					
100	0.582	1.082	-0.067	1.618	0.698	0.701	0.021	64
	(0.695)	(0.686)	(0.818)	(0.403)	(0.033)	(0.032)	(0.021)	
200	0.505	1.051	0.015	1.565	0.701	0.699	0.02	93.3
	(0.278)	(0.314)	(0.407)	(0.269)	(0.024)	(0.024)	(0.016)	
500	0.510	1.007	-0.006	1.521	0.7	0.7	0.02	100
(0.176)	(0.188)	(0.252)	(0.160)	(0.015)	(0.014)	(0.009)		
**Dominant**			***α*_2_ = 1**					
100	0.586	1.089	1.008	1.643	0.698	0.701	0.021	74.8
	(0.718)	(0.710)	(0.887)	(0.468)	(0.033)	(0.033)	(0.021)	
200	0.523	1.038	1.013	1.557	0.701	0.7	0.02	96.4
	(0.279)	(0.308)	(0.423)	(0.284)	(0.023)	(0.023)	(0.015)	
500	0.506	1.009	1.002	1.518	0.7	0.7	0.02	100
	(0.169)	(0.191)	(0.265)	(0.166)	(0.015)	(0.014)	(0.009)	
**Recessive**			***α*_2_= -1**					
100	0.581	1.098	-1.119	1.622	0.699	0.699	0.019	88
	(0.789)	(0.798)	(0.919)	(0.418)	(0.032)	(0.034)	0.022	
200	0.519	1.040	-1.036	1.572	0.7	0.699	0.02	97.6
	(0.287)	(0.316)	(0.421)	(0.269)	(0.023)	(0.023)	0.015	
500	0.504	1.016	-1.013	1.530	0.7	0.699	0.02	100
	(0.186)	(0.182)	(0.261)	(0.164)	(0.015)	(0.015)	(0.009)	

$p_{1}^{\left(1\right)}$ ,  $p_{1}^{\left(2\right)}$ and *D* are the allelic frequencies of alleles $Q_{1}^{1}$ and $Q_{1}^{2}$  at two SNPs and their linkage disequilibrium, respectively. *α*_0_  is the intercept, and *α*_1_  and *α*_2_  are the additive and dominant effects respectively by assuming that haplotype $\left[Q_{1}^{1}Q_{1}^{2}\right]$ is different from the rest haplotypes. *γ* is the covariate effect. The first row contains the given values of all parameters. Power is calculated as the percentages of all simulations in which the true disease-gene association is detected.

**Table 4.** The Mean MLEs with their Square Root Mean Square Errors (RMSEs) (in Parentheses) of Population and Genetic Parameters of the BTNs Estimated from 1000 Simulation Replicates Under the 3-SNP Model

*n*	*α*_0_	*α*_1_	*α*_2_	*γ*	$p_{1}^{\left(1\right)}$	$p_{1}^{\left(2\right)}$	$p_{1}^{\left(3\right)}$	*D*_12_	*D*_13_	*D*_23_	*D*_123_	Power
True*	0.5	1.0		1.5	0.7	0.7	0.7	0.04	0.025	0.025	0.02	
**Additive**			***α*_2_** = 0									
100	0.622	1.156	-0.117	1.614	0.698	0.699	0.699	0.04	0.025	0.024	0.02	68.8
	(0.998)	0.992)	(1.076)	(0.405)	(0.033)	(0.033)	(0.033)	(0.020)	(0.021)	(0.021)	(0.013)	
200	0.542	1.038	-0.028	1.547	0.699	0.700	0.699	0.04	0.026	0.024	0.02	93.1
	(0.307)	(0.311)	(0.413)	(0.245)	(0.023)	(0.023)	(0.024)	(0.014)	(0.015)	(0.015)	(0.009)	
500	0.504	1.013	-0.004	1.522	0.700	0.700	0.699	0.04	0.025	0.024	0.02	100
	0.179)	0.186)	(0.256)	(0.159)	(0.015)	(0.015)	(0.016)	(0.009)	(0.009)	(0.009)	(0.006)	
**Dominant**			***α*_2_** = 1									
100	0.677	1.201	0.975	1.654	0.700	0.699	0.699	0.04	0.025	0.024	0.02	83.2
	(1.276)	(1.302)	(1.566)	(0.480)	(0.033)	(0.033)	(0.033)	(0.020)	(0.021)	(0.020)	(0.013)	
200	0.544	1.036	0.997	1.547	0.699	0.700	0.699	0.04	0.026	0.024	0.02	99.2
	(0.305)	(0.318)	(0.459)	(0.274)	(0.023)	(0.023)	(0.024)	(0.014)	(0.015)	(0.015)	(0.009)	
500	0.506	1.012	1.013	1.521	0.700	0.700	0.699	0.04	0.025	0.024	0.02	100
	(0.178)	(0.190)	(0.281)	(0.169)	(0.015)	(0.015)	(0.015)	(0.009)	(0.009)	(0.009)	(0.006)	
**Recessive**			***α*_2_** = -1									
100	0.637	1.18	-1.151	1.631	0.702	0.701	0.702	0.04	0.025	0.024	0.02	89.3
	(1.128)	(1.15)	(1.217)	(0.429)	(0.033)	(0.033)	(0.032)	(0.021)	(0.021)	(0.021)	(0.013)	
200	0.52	1.032	-1.025	1.552	0.699	0.7	0.7	0.04	0.026	0.024	0.02	97.8
	(0.299)	(0.296)	(0.438)	(0.262)	(0.024)	(0.023)	(0.023)	(0.014)	(0.015)	(0.015)	(0.009)	
500	0.502	1.01	-1.01	1.521	0.700	0.700	0.699	0.04	0.025	0.024	0.02	100
	(0.183)	(0.193)	(0.262)	(0.153)	(0.015)	(0.015)	(0.015)	(0.009)	(0.009)	(0.009)	(0.006)	

$p_{1}^{\left(1\right)}$ , $p_{1}^{\left(2\right)}$ and $p_{1}^{\left(3\right)}$ are the allelic frequencies of alleles $Q_{1}^{1}$ , $Q_{1}^{2}$ and  $Q_{1}^{3}$ at three SNPs, and  *D*_12_ , *D*_23_ , *D*_13_ , and  *D*_123_ are their linkage disequilibrium, respectively by assuming that haplotype ${[Q}_{1}^{1}Q_{1}^{2}Q_{1}^{3}]$ is different from the rest haplotypes. See Table 3 for explanations of other parameters.

**Table 5.** The Maximum Likelihood Estimates (MLEs) of the Population and Quantitative Parameters for Significant BTNs Associated with LGA Detected within *APOC3* Gene. The Standard Errors of the Quantitative Parameters are Given in the Parenthesis

		AIC	LR_1_	*P* - value
Risk haplotype	[TC]	**558.2**	**7.429**	**0.024**
	[TG]	565.29	0.336	0.845
	[CG]	-	-	-
	[CC]	562.68	2.948	0.229
***Population parameters***				
Haplotype frequencies	${\hat{p}}_{\mathrm{TG}}$	0.6196		
	${\hat{p}}_{\mathrm{TC}}$	0.1259		
	${\hat{p}}_{\mathrm{CG}}$	0		
	${\hat{p}}_{\mathrm{CC}}$	0.2545		
Allele frequencies and LD	${\hat{p}}_{T}^{1}$	0.7455		
	${\hat{p}}_{C}^{2}$	0.3804		
	$\underline{\hat{D}}$	-0.1577		
***Quantitative parameters***				
Intercept	${\hat{\alpha }}_{0}$	-3.235(0.851)		
Additive effect	${\hat{\alpha }}_{1}$	-0.606(0.547) ^**^		
Dominant effect	${\hat{\alpha }}_{2}$	-0.092(0.612) ^**^		
MA	${\hat{\gamma }}_{1}$	0.036(0.016) ^*^		
MW	${\hat{\gamma }}_{2}$	0.043(0.022)^*^		
PTD	${\hat{\gamma }}_{3}$	-0.328(0.281)		
BS	${\hat{\gamma }}_{4}$	-0.547(0.215)^**^		
MBMI	${\hat{\gamma }}_{5}$	-0.076(0.056)		

LR_1_ is the likelihood ratio test statistic based on hypothesis (12). The risk haplotype detected on the basis of the AIC value and LR test is indicated in boldface.** and * refer to significance at the 0.01 and 0.05 level, respectively.

MA=maternal age; MW=maternal weight; PTD=number of preterm deliveries; BS=baby sex; MBMI=maternal body mass index.

## APPENDIX

### EM ALGORITHM FOR ESTIMATING THE PARAMETERS

The EM algorithm for the 2-SNP model is detailed here, while the EM algorithm for the *R* -SNP (R ≥ 3 ) model is similar and hence is omitted.

Let *y_1_*  be the response vector containing the disease status for individual *i* $\left(i=1,\cdot \cdot \cdot ,n.\text{ with }n.=n-n_{12/12}\right)$ , *y_0_*  be the response vector for individual  *i*$\left(i=1,\cdot \cdot \cdot ,n_{12/12}\right)$ who carries genotype 12/12. Let *X_2_*  be a matrix corresponding to response vector *y_1_*  with the first column being all ones, the second and third column being the elements of  *x_i1_* and *x_i2_*  defined in Equation (4) and (5) for individual *i* respectively and the rest columns containing all the non-genetic covariates. Denote  *X_1_*  a matrix with its first and third column being ones, second column being zeros, and similarly denote  *X_0_*  a matrix with first column being ones, second column being negative ones, third column being zeros, and the rest columns being the non-genetic covariates corresponding to individuals with genotype 12/12. Therefore, the dimension of  *X_2_*  is *n.* × (*p* + 3) , and the dimension of  *X_1_* and  *X_0_* is  *n_12/12_* × (*p* + 3) where  *p* is the number of non-genetic covariates.

Let *c_i_*  be the diplotype of the BTNs with observed genotype 12/12 ( *c_i_* = 1  if diplotype [11][22] and = 0 if diplotype [12][12]). Since  *c_i_* is unobservable, it is treated as missing data. Define the following three logistic regression functions

$\pi_{2i}=\pi \left(y_{\mathrm{1i}}=1|X_{2i}\right),\pi_{\mathrm{1i}}=\pi \left(y_{0i}=1|X_{\mathrm{1i}}\right)\text{ and }\pi_{0i}=\pi \left(y_{0i}=1|X_{0i}\right)$

where *y_0i_* and *y_1i_* are the observations corresponding to double heterozygous and other genotypes, respectively. Then, the complete data log-likelihood function is given by

$\begin{array}{c} \begin{array}{cc} l_{n}^{c}\left(\beta \right)= & \sum_{i=1}^{\mathrm{n1}}\text{log}\left[\pi_{2i}^{\mathrm{yli}}{\left(1-\pi_{2i}\right)}^{1-\mathrm{y1i}}\right] \end{array}\\ +\sum_{i=1}^{\mathrm{n0}}\left\{c_{i}\text{log}\left[\pi_{\mathrm{li}}^{\mathrm{y0i}}{\left(1-\pi_{\mathrm{li}}\right)}^{1-\mathrm{y0i}}\right]+\left(1-c_{i}\right)\text{log}\left[\pi_{0i}^{\mathrm{y0i}}{\left(1-\pi_{0i}\right)}^{1-\mathrm{y0i}}\right]\right\}\\ =-\sum_{i=1}^{\mathrm{n1}}\left[1+\exp\left(\sum_{j=0}^{p}\beta_{j}x_{2\mathrm{ij}}\right)\right]+\sum_{i=1}^{\mathrm{n1}}y_{\mathrm{li}}\sum_{j=0}^{p}\beta_{j}x_{2\mathrm{ij}}\\ +\sum_{i=1}^{\mathrm{n0}}\left\{c_{i}\text{log}\left[\pi_{\mathrm{li}}^{\mathrm{y0i}}{\left(1-\pi_{\mathrm{li}}\right)}^{1-\mathrm{y0i}}\right]+\left(1-c_{i}\right)\text{log}\left[\pi_{0i}^{\mathrm{y0i}}{\left(1-\pi_{0i}\right)}^{1-\mathrm{y0i}}\right]\right\} \end{array}$

Thus, in the E-step of the (*t*)th EM iteration, we only need to calculate

(A1) $$\begin{array}{c} \begin{array}{ccc} {\bar{\omega }}_{i}^{\left(t\right)}= & E\left[c_{i}\left|y_{0i},X_{1i},X_{0i},\phi ,\beta^{\left(t\right)}\right.\right]= & P\left(c_{i}=1\left|y_{0i},X_{1i},X_{0i},\phi ,\beta^{\left(t\right)}\right.\right) \end{array}\\ =\frac{{\phi \left[\pi_{\mathrm{1i}}^{\left(t\right)}\right]}^{\mathrm{y0i}}{\left[\left(1-\pi_{\mathrm{1i}}^{\left(t\right)}\right)\right]}^{1-\mathrm{y0i}}}{{\phi \left[\pi_{\mathrm{1i}}^{\left(t\right)}\right]}^{\mathrm{y0i}}{\left[\left(1-\pi_{\mathrm{1i}}^{\left(t\right)}\right)\right]}^{1-\mathrm{y0i}}+\left(1-\phi \right){\left[\pi_{0i}^{\left(t\right)}\right]}^{\mathrm{y0i}}{\left[\left(1-\pi_{0i}^{\left(t\right)}\right)\right]}^{1-\mathrm{y0i}}} \end{array}$$

Replace the missing value *c_i_*  by ${{\bar{\omega }}_{i}}^{\left(t\right)}$ in the log-likelihood function with the complete data and then in the M-step, we maximize

$\begin{array}{c} \begin{array}{cc} Q^{\left(t\right)}= & -\sum_{i=1}^{n_{1}}\left[1+\exp\left(\sum_{j=0}^{p}\beta_{j}x_{2\mathrm{ij}}\right)\right]+\sum_{i=1}^{n_{1}}\mathrm{y1i}\sum_{j=0}^{p}\beta_{j}x_{2\mathrm{ij}} \end{array}\\ +\sum_{i=1}^{\mathrm{n0}}\left\{{\bar{\omega }}_{i}^{\left(t\right)}\text{log}\left[\pi_{1i}^{\mathrm{y0i}}{\left(1-\pi_{1i}\right)}^{1-\mathrm{y0i}}\right]+\left(1-{\bar{\omega }}_{i}^{\left(t\right)}\right)\text{log}\left[\pi_{0i}^{\mathrm{y0i}}{\left(1-\pi_{0i}\right)}^{1-\mathrm{y0i}}\right]\right\} \end{array}$

with respect to β. To do so, we can use the Newton-Raphson iteration method which needs the first and the second partial derivatives given below.

$\begin{array}{cc} \frac{\partial Q^{\left(t\right)}}{\partial \beta_{j}}= & -\sum_{i=1}^{n_{1}}x_{2\mathrm{ij}}\pi_{2i}+\sum_{i=1}^{n_{1}}y_{\mathrm{li}}x_{2\mathrm{ij}}+\sum_{i=1}^{n_{0}}\left[x_{1\mathrm{ij}}{\bar{\omega }}_{2i}^{\left(t\right)}\left(y_{0i}-\pi_{1i}\right)+x_{0\mathrm{ij}}{\bar{\omega }}_{i}^{\left(t\right)}\left(y_{0i}-\pi_{0i}\right)\right]\\ \frac{\partial^{2}Q^{\left(t\right)}}{\partial \beta_{j}\beta_{k}}= & -\sum_{i=1}^{n_{1}}x_{2\mathrm{ij}}x_{2\mathrm{ik}}\pi_{2i}\left(1-\pi_{2i}\right)-\sum_{i=1}^{n_{0}}\left[x_{1\mathrm{ij}}x_{1\mathrm{ik}}{\bar{\omega }}_{i}^{\left(t\right)}\pi_{1i}\left(1-\pi_{1i}\right)+x_{0\mathrm{ij}}x_{0\mathrm{ik}}{\bar{\omega }}_{i}^{\left(t\right)}\pi_{0i}\left(1-\pi_{0i}\right)\right] \end{array}$

The Hessian matrix at the (*t*) th iteration is given by $H^{\left(t\right)}=\frac{\partial^{2}Q^{\left(t\right)}}{\partial \beta_{j}\beta_{k}}$ which leads to the updated parameters β at the (*t*+1) th iteration

$\begin{array}{cc} \beta^{\left(t+1\right)}= & \beta^{\left(t\right)}-{\left[H^{\left(t\right)}\right]}^{-1}u^{\prime } \end{array}$

Where *u* is a vector of first derivative of *Q^(t)^*  with respect to *β_j_* . The EM algorithm is repeated between Equation (A1) and (A2) until certain convergence criteria is satisfied. One of the by-product of using Newton-Raphson method is that we can easily obtain the standard errors of the estimated parameters through the Hessian matrix.
